# The cost of diabetes in Latin America and the Caribbean in 2015: Evidence for decision and policy makers

**DOI:** 10.7189/jogh.07.020410

**Published:** 2017-12

**Authors:** Alberto Barcelo, Armando Arredondo, Amparo Gordillo–Tobar, Johanna Segovia, Anthony Qiang

**Affiliations:** 1 University of Miami, Miller School of Medicine, Miami, Florida, USA; 2 National Institute of Public Health, Cuernavaca, Mexico; 3 World Bank, Washington D.C., USA; 4 McMaster University, Hamilton, Canada

## Abstract

**BACKGROUND:**

The financial implications of the increase in the prevalence of diabetes in middle–income countries represents one of the main challenges to health system financing and to the society as a whole. The objective of this study was to estimate the economic cost of diabetes in Latin America and the Caribbean (LAC) in 2015.

**METHODS:**

The study used a prevalence–based approach to estimate the direct and indirect costs related to diabetes in 29 LAC countries in 2015. Direct costs included health care expenditures such as medications (insulin and oral hypoglycemic agents), tests, consultations, hospitalizations, emergency visits and treating complications. Two different scenarios (S1 and S2) were used to analyze direct cost. S1 assumed conservative estimates while S2 assumed broader coverage of medication and services. Indirect costs included lost resources due to premature mortality, temporary and permanent disabilities.

**RESULTS:**

In 2015 over 41 million adults (20 years of age and more) were estimated to have Diabetes Mellitus in LAC. The total indirect cost attributed to Diabetes was US$ 57.1 billion, of which US$ 27.5 billion was due to premature mortality, US$16.2 billion to permanent disability, and US$ 13.3 billion to temporary disability. The total direct cost was estimated between US$ 45 and US$ 66 billion, of which the highest estimated cost was due to treatment of complications (US$ 1 616 to US$ 26 billion). Other estimates indicated the cost of insulin between US$ 6 and US$ 11 billion; oral medication US$ 4 to US$ 6 billion; consultations between US$ 5 and US$ 6 billion; hospitalization US$ 10 billion; emergency visits US$ 1 billion; test and laboratory exams between US$ 1 and US$ 3 million. The total cost of diabetes in 2015 in LAC was estimated to be between US$ 102 and US$ 123 billion. On average, the annual cost of treating one case of diabetes mellitus (DM) in LAC was estimated between US$ 1088 and US$ 1818. Per capita National Health Expenditures averaged US$ 1061 in LAC.

**CONCLUSIONS:**

Diabetes represented a major economic burden to the countries of Latin America and the Caribbean in 2015. The estimates presented here are key information for decision–making that can be used in the formulation of policies and programs to achieve greater efficiency and effectiveness in the use of resources for diabetes prevention in the 29 countries of LAC.

The cost of diabetes mellitus (DM) was estimated at US$ 65 billion in 2000 in Latin America and the Caribbean (LAC) [1]. This burden was attributed to lost productivity due to mortality and disability, as well as direct medical costs caused by treating diabetes and its long–term complications.

The financial implications of the increase in the prevalence of diabetes in middle–income countries, represents one of the main challenges to be solved by health systems and society as a whole. DM is one of the top global public health concerns because of the increased morbidity and mortality among affected individuals. An estimated 415 million people suffer from diabetes globally in 2015 and this number is expected to reach the 642 million mark by 2040 [2]. This will increase direct health expenses as well as premature deaths and disability to unprecedented levels unless effective preventive and control strategies are implemented.

DM presents a global public health challenge, particularly among middle and low–income countries, where 80% of those with the disease live. Diabetes management is complex because it demands continuous care involving many services, tests, and medications, owing to the detrimental effect of chronic hyperglycemia on organs and tissues.

According to the 2012 World Health Organization (WHO) National Capacity Survey for Non Communicable Diseases (NCD) Prevention and Control, 95% of countries in the Americas reported having DM policies or action plans. However, the capacity to prevent and control diabetes may be limited. In fact, only one third of the countries reported having operational evidence–based guidelines or protocols [3]. Population–based surveys and clinical series from many of the countries of LAC indicated that most people with diagnosed DM do not achieve treatment goals [4–9], increasing the risk of premature mortality and disabilities.

The objective of this study was to estimate the economic cost of diabetes in LAC in 2015. This study intends to update a previous estimate of the cost of diabetes in LAC for 2000 [1].

## METHODS

This study used a prevalence–based approach that includes direct and indirect costs caused by diabetes. Adult (20 and more years of age) diabetes prevalence data for 2015 were used to estimate the direct and indirect costs related to diabetes in 29 LAC countries (Latin America: Argentina, Bolivia, Brazil, Chile, Colombia, Costa Rica, Cuba, Dominican Republic, Ecuador, El Salvador, Guatemala, Honduras, Mexico, Nicaragua, Panama, Paraguay, Peru, Puerto Rico, Uruguay and Venezuela; English Caribbean: Bahamas, Barbados, Belize, Grenada, Guyana, Jamaica, Saint Lucia, Suriname and Trinidad & Tobago). Estimates included people with both type 1 and type 2 diabetes. The protocol for this project was reviewed and approved by the Committee on Health Research of the National Council of Science and Technology of Mexico. Patient records information was anonymized and de–identified prior to analysis.

### Data

Direct costs included insulin and oral hypoglycemic agents, consultations, A1c tests, lipid profiles, micro albuminuria test, hospitalizations, emergency visits and treating complications. A “bottom–up” approach was used for direct cost estimation.

Indirect costs included lost resources due to mortality, as well as temporary and permanent disabilities. These were estimated using the human capital approach, which applies the present value of future earnings to estimate the burden of mortality and disability [10].

LAC demographic data for 2015 [11] were combined with prevalence data for 2015 from the International Diabetes Federation (IDF) to estimate the population with diabetes by age and gender for each country. Countries were classified using the World Bank GDP per capita classification [12] in low–income countries (US$ 1035 or less), lower middle–income countries (US$ 1036 to US$ 4085), upper middle–income countries (US$ 4086 to US$ 12 615), and high–income countries (US$ 12 616 or more) [13]. The most recent National Health Expenditures (NHE) for each country were obtained from the Pan American Health Organization (PAHO) basic data indicators [14]. NHE for Puerto Rico was not available.

Five sources of original country data were used to produce estimates:

1. Commercial prices reported to the NCD Management National Capacity survey for chronic diseases in LAC [15] were obtained for tests, medications, and medical services. Prices were updated in 2015 by contacting health professionals and diabetes associations in each country. The PAHO database contains prices for insulin, oral medication, testing and services for 26 out of the 29 countries [15]. When a specific price for one country was not available we used an average price for countries of the same sub region (English Caribbean, Central or South America). The price list is available in **Online Supplementary Document(Online Supplementary Document)**.

2. The Sentinel Surveillance (ViCen) questionnaire was completed for a random sample of 1 899 patients in clinics in six countries (Bolivia, Chile, El Salvador, Guatemala, Honduras and Nicaragua). This survey explored health care utilization along with the presence of DM and diabetes–related long–term complications. The ViCen questionnaire was produced by PAHO in collaboration with the IDF and was used in previous research in Brazil [16,17].

3. A clinical record database of 51 795 patients from Chile was used to calculate the prevalence of chronic complications (retinopathy, nephropathy, cardiovascular disease, neuropathy and peripheral vascular disease) [18]. This database was also used to produce estimates of permanent disability by age and sex adjusted by diabetes duration.

4. A database from the National Institute of Public Health (NIPH) in Mexico was used to estimate the cost of cases with and without long–term diabetes complications. The NIPH database provided an estimation of how much higher was the cost of cases with each long–term diabetes complication as compared to treating cases without complications [19].

5. The Barbados Eye Study (BES) is a cohort study funded by the National Eye Institute. It is a nationally representative cohort of 4 709 Barbados–born citizens, ages 40 to 84 years and selected by simple random sampling. BES used Relative Risk Ratios (RR), based on the hazard ratios estimated from the Cox Proportional Hazard Model. RR was calculated comparing mortality for people with and without diabetes by age and gender using 9–year follow–up data (1997–2002) [20].

### Direct costs

Two analyses were conducted separately to calculate direct cost. These analyses are identified as Scenario 1 (S1) and Scenario 2 (S2). S1 and S2 differ in estimations for the use of medication and the cost increase related to treatment of diabetes chronic complications. S1 contains conservative estimates while S2 assumes broader coverage of medication and services. Assumptions for S1 and S2 are described below. A sensitivity analysis was conducted to compare results of the cost for S1 and S2.

### Medication

**Insulin**. The annual total cost of insulin (TCI) for the 29 LAC countries was estimated as follows:

TCI = NIU × CI × IC(1)

where NIU is the number of insulin users, CI is the cost of insulin per unit in each country, and IC is the number of insulin unit consumption per year.

Patterns of insulin use might be different in each country. Population based research indicated that among people with diabetes insulin use ranged from 2% in Chile [4] and 29% in Costa RicaRica [7]. Due to the paucity of information regarding the heterogeneity in the use of insulin use, we estimated two different proportion of insulin use: 10% (S1) and 20% (S2) of the diabetes population of each country.

Insulin consumption was assumed at 10 000 IU per person per year, as previously used by Phillips and Salmeron [21] and used in our previous analysis also [1]. The commercial cost of insulin in each country was taken from the PAHO database price list [15].

**Oral hypoglycemic agents**. The annual total cost of oral medications (TCO) for the 29 countries studied was estimated as follows.

TCO = NOU × CO × OC(2)

where NOU is the number of oral medication users, CO is the cost of oral medications (metformin) in each country extracted from the PAHO database, and OC is the estimated number of pills consumed per person each year.

The proportion of people taking oral medication varies from 90% in Cuba [22] to 50% in Belize [7]. As the proportion of people with diabetes taking oral medication might vary among countries two different assumptions were applied. We assumed that 50% or 80% of people with diabetes were taking oral medication for S1 and S2, respectively. The per capita consumption of metformin each year was estimated at 1 500 tablets. Prices of Metformin 800 mg were taken from the PAHO database [15].

### Consultations and hospitalizations

The total number of consultations and hospitalizations in the general population of each country was obtained from the PAHO basic indicator database [14]. These consultations and hospitalizations were considered to be due to general causes not related to diabetes.

The ViCen survey showed that people with diabetes made 3.5 times more medical visits and had 7 times more hospitalizations than people without diabetes [16]. In addition, the ViCen study showed that length of stay for those with diabetes was 1.9 days longer than those without diabetes [16]. The numbers of medical visits and hospitalization per inhabitants in each country were multiplied by factors of 2.55 and 6, respectively, which represented the excess number of visits and hospitalizations due to diabetes [6,7]. The estimated number of people with diabetes was then multiplied by these products. In the case of hospitalizations, we obtained the total number of hospital days by multiplying the number by the average of length of stay in each country reported to the PAHO basic indicator database [14], and then increased by the fraction of 0.9 which was the excess number of days attributed to diabetes reported by patients to the ViCen study [16]. The costs of consultations and per–day hospitalization were obtained from PAHO’s price list [15]. The cost of services, procedures or treatment was not included due to the paucity in country –specific data.

The annual total cost of consultations (TCC) was estimated as follows:

TCC = [(NC × CC) × COC] × DM(4)

where NC is the number of consultations per year, CC is the average cost of a general medicine consultation in each country, COC is the average cost of a consultation with an ophthalmologist in each country, and DM is the number of people under care. It was assumed that, across the region, 55% of those with DM were treated by a health care providers.

The annual total cost of hospitalizations (TCH) was estimated as follows:

TCH = NH × LH × CH × DM(5)

where NH is the annual number of hospitalizations, LH is the length of stay, CH is the average cost of a hospitalization, and DM is the number of those with diabetes who were at risk of being hospitalized. It was assumed that the total number of those with DM was at risk of being hospitalized.

The costs of hospitalizations related to DM and for general causes were calculated separately. Only the excess cost attributed to DM was included in the direct cost calculation. The cost of one day hospital stay was taken from the PAHO’s price list [15]. This cost does not include any service or procedure. This cost is assumed to be conservative since it is well known that most cases of diabetes would require specific services and specialized care which are usually more expensive.

The average cost of a general and ophthalmologist medical visit per country was taken from the PAHO database [15].

The cost of medical visits related to DM and for general causes were calculated separately; only the excess cost estimated for those consultations attributed to DM was included in the direct cost calculation. The cost of consultation to a general practitioner in each country was taken from PAHO’s price list [15] and was used as a proxy for all DM medical consultations because country specific prices for specialist visits (except for ophthalmologist) were not available. This cost does not include any additional procedures, it includes only a consultation with a physician.

### Testing

The annual total cost of examinations (TCE) for the 29 countries was estimated as follows:

TCE = (A1c × Lipid + EKG + Rx + Protein) × DM(3)

where A1c is the cost of a glycated hemoglobin test, Lipid is the cost of a lipid profile, EKG is the cost of an electrocardiogram, Rx is the cost of a chest x–ray, protein is the cost of a urine protein test, and DM is the total population with diabetes.

It was assumed that each person with DM was tested once a year with lipid profile, one electrocardiogram, one chest x–ray, and one urine protein test. The number of A1c test was assumed to be 1 per year for S1 and 3 per year for S2.

### Emergency visits

The annual total cost of emergency visits (TCEV) was estimated as follows:

TCEV = NEC × CEV × DM(6)

where NEC is the average annual number of emergency visits, CEV is the average cost of an emergency visit in each country taken from the PAHO price list, and DM is the total number of people with diabetes in each country.

The ViCent survey showed that those with diabetes made 1.4 emergency visit per year while those without diabetes reported 0.9 visits per year [16]. The estimated number of people with diabetes was multiplied by 1.4 to estimate the number of emergency visit. The cost of an emergency department visit in each country was obtained from the PAHO’s price list [15].

The emergency cost does not include the cost of any procedure, it includes only the cost of being seen by a physician in an emergency department. The cost of emergency visits due to general causes was discounted. Only emergency visits attributed to diabetes (0.5 per person per year) were included.

### Complications

This study included the cost of the following complications: cardiovascular disease, nephropathy, neuropathy, peripheral vascular disease and retinopathy. It also included the costs of dialysis, hemodialysis, photocoagulation and treating foot–related problems.

Three steps were used to estimate the cost of complications. First, the weighted probability of major complications from the Chilean QUALIDIAB [18] database were applied to the diabetes population to obtain the number of persons with each complication per country. Second, we estimated the cost of regular care for uncomplicated cases of DM for each country. We made different assumptions for regular care for S1 and S2. Population based surveys showed wide variation in the proportion of cases of diabetes under care in different countries. Therefore, we made different assumptions for the two scenarios. For S1 regular care for uncomplicated cases one visit to an ophthalmologist, one A1c, one lipid profile, one urine protein test, one electrocardiogram and one chest x–ray for 55% of the population with diabetes. This was a conservative estimate that was based on protocols available in some of the participating countries. For S2 regular care for uncomplicated cases included one visit to an ophthalmologist, three A1c, one lipid profile, one urine protein test, one electrocardiogram and one chest x–ray for 85% of the population with diabetes.

Available data from Chile [1], Brazil [17], and Mexico [19] showed that the ratio of expenditure per person with diabetes and chronic complications to persons with diabetes without complications was variable. Because of the heterogeneity of health care provision and medical costs across countries, for each complication a lower (S1) and higher (S2) ratio was used to produce two separate estimates. Among the three sources, we selected the lowest estimated cost ratio (S1) and an average of the other two estimates (S2). The ratio used for these calculations were retinopathy 1.09 and 1.86; neuropathy 1.08 and 1.58; Peripheral Vascular Disease 1.06 and 1.81; Cardiovascular Disease 1.23 and 1.86; and Nephropathy 1.76 and 2.96 for S1 and S2 respectively.

Third, we applied the excess of cost of treating (consultations, medications and other procedures) for each complication as per S1 and S2 cost ratio using the cost estimates for uncomplicated cases in each country as per previous calculation.

The total cost of consultations for retinopathy (TCCR) was estimated as follows:

TCCR = [(NCR × CC) + COC] × DM × PVR (7)

where TCCR is the annual cost of consultations for those with diabetes retinopathy, NCR is the number of consultations per person with DM–related retinopathy, and PVR is the prevalence of retinopathy.

The total cost of consultations for other (TCCO) DM–related complications was estimated as follows:

TCCO = ∑*_i∈I_* [[((NCC*_i_* ]]× CC) + COC) × DM × PV*_i_* (8)

where TCCO is the annual cost of consultations for those with DM and long–term complications other than retinopathy, NCC is the number of consultations per person with a DM–related complication other than retinopathy, PV is the prevalence of the complication, and *i* represents the four complications studied, excluding retinopathy.

The total cost of consultations for complications (TCCC) of those with DM–related complications was estimated as follows:

TCCC = TCCR + TCCO (9).

The total cost of hospitalizations related to complication (TCHC) was estimated as follows:

TCHC = ∑*_i∈I_* NHC*_i_* × LHC*_i_* × CH × DM × PV*_i_*  (10)

where TCHC is the annual cost of hospitalizations for those with DM–related complications, NHC is the number of hospitalizations by person with DM–related complications, LHC is the length of a patient’s hospital stay, CH is the average cost of a hospitalization per person, PV is the prevalence of complications, and i is the set of complications studied.

The used age–weighted probability for each long–term complication was 1.09 for retinopathy, 1.23 for CVD, 2.35 for nephropathy, 1.08 for neuropathy, and 3.35 for PVD [18].

### Indirect costs

#### Mortality costs

Mortality costs were estimated by multiplying the number of DM–related deaths by age range and gender by the number of years of productive life lost (YPLL) for each age by the country GDP per capita. A three percent discount was applied.

The number of DM–related deaths was calculated by multiplying the number of deaths for all causes, by the population attributable–fraction (PAF), which was estimated as follows:

PAF = P(RR–1)/1 + [P(RR–1)](11)

where P is the prevalence of DM and RR is the relative risk of death from the disease.

The Barbados Eye Study (BES) is a cohort study that includes RR obtained from the Barbados Eye Study, which contained information from a cohort of people with diabetes. The RR used were Men 40–49 = 3.4 (1.2–9.3); 50–59 = 2.0 (1.0–3.7); 60–64 = 3.2 (2.2–4.7) and Women 40–49 = 2.8 (1.2–6.4); 50–59 = 4.4 (2.4–8.3); 60–64 = 2.3 (1.1–1.9) (19). YPLL were included for individuals dying between the ages of 40 and 64 years. Only deaths estimated to occur between the ages of 40 and 79 years of age were included. YPLL between the ages of 40 and 64 years of ages were calculated since the age of retiring was assumed to be 65 years of age. 95%–Confidence intervals were calculated by applying lower and higher estimates for the OR of original BES data [19].

#### Disability costs

**Permanent disability.** The total proportion of people with disability was estimated at 4.2% of the total target population. After discounting cases considered to be related to general causes, we estimated that 3.2% of the target population was disable due to diabetes. Estimates for other diseases and diabetes related disability by age and gender were obtained by applying research results from Gomez de Moura et al [23] to Brazil’s national statistics [24]. The probability of diabetes related disability by age and gender was applied to the Chilean database and adjusted throughout a logistic regression by diabetes duration and the presence of chronic diabetes complications that would prevent people to remain in the workforce. These complications were blindness, amputations, strokes and infarction. The age–and–gender specific estimates were multiplied by the number of people with diabetes by age and gender to obtain the number of disabled due to diabetes. We used these estimates to calculate the number of discounted years of productive life lost (YPLL) before the age of 65 years. Only one year of income lost per person was included. GNP per capita for each country was used as a proxy for lost productivity in 2015. The cost of permanent disability was estimated by multiplying the per capita GNP by the number of YPLL.

**Temporary disability.** Only absenteeism was included as cost of temporary disability. The lack of information on other aspects of temporary disability such as bed days due to illness or care takers and others were not included. Temporary disability costs were calculated by multiplying the number of economically active people with DM by age and gender between the ages of 20 and 64 years, by the number of days lost due to disability by national annual GDP per capita (divided by 365) as a proxy of potential income per day. The number of people that were economically active in each country was calculated by discounting the unemployed as per data published by the World Bank [12]. The number of lost productive days was calculated with data from the ViCen survey [16]. The estimated number of lost days for general causes not related to DM was subtracted from the total lost days. Thus, the number of lost days included were only the excess number attributed to DM.

All costs estimates are presented in 2015 US dollars ($).

95%–CI for permanent/ temporary disability as well for indirect cost, were calculated by using bootstrap analysis with 1000 replications of age–and–gender specific estimates.

All analysis were performed using SPSS 24 (IBM Analytics, Armonk, New York, USA) and Stata 12 (StataCorp, College Station, Texas, USA).

## RESULTS

Over 41 million people were estimated to have DM throughout LAC in 2015. From the total in LAC of those with DM, approximately 97% lived in Latin America (Central–South America, Mexico and the Spanish Caribbean) and 2% in the English Caribbean. Overall, among components of indirect cost, US$ 27.5 billion was attributed to diabetes related premature mortality, US$ 16.2 billion to permanent disability, and US$ 13.3 billion to temporary disability. The total indirect cost was US$ 57.2 (US$ 54.9–60.4) billion (**Table 1**).

**Table 1:** Data on those with diabetes mellitus (DM) and estimated indirect costs in Latin America and the Caribbean, 2015

Item	Latin America	English Caribbean	Total
Populations 20–79 (×10^3^)	315 188	2598	317 786
Total No. of people with diabetes (×10^3^)	41 022	554	41 576
**Mortality:**			
No. of deaths	1 364 376	22 137	1 386 513
Deaths related to diabetes (40–64 years)	313 205	4897	318 102
YPLL	2 044 418	30 214	2 074 633
Cost (US$×10^6^)	27 280	305	27 585
95% CI (US$×10^6^)	8 066–47 905	90–570	8157–48 475
**Permanent disability:**			
Total No. of people with permanent disability	1 389 057	17 407	1 406 464
YPLL due to DM	1 016 571	26 557	1 043 128
Cost in 1 year (US$×10^6^)	15 863	342	16 205
95% CI (US$×10^6^)	15 115–16 702	316–517	15 450–17 037
**Temporary disability:**			
Estimated DM employed population (20–64)	31 463 016	409 686	31 872 702
No. of days missed of work general causes	665 563 904	8 306 850	673 870 754
No. of days missed of work due to DM	309 631 903	3 864 491	313 496 394
YPLL	847 567	10 588	858 154
Cost (US$×10^6^)	13 212	179	13 391
95% CI (US$×10^6^)	12 773–13 735	170–191	12 944–13 906
Indirect cost (US$×10^6^)	56 355	826	57 181
95% CI (US$×10^6^)	54 045–59 565	799–992	54 881–60 411

YPLL – years of productive life lost; CI – confidence interval

**Table 2** presents estimates of the cost of medication as well as exams and consultations for scenario 1 (S1) and scenario 2 (S2). Overall 4 to 6 million people with diabetes were estimated to use insulin with an estimated cost between US$ 6.9 to US$ 11.6 billion. The number of oral medication users was estimated between 19.7 and 33.5 million with an estimated cost of US$ 11 to US$ 18 million. The total cost of medication was estimated to be between US$ 11 to US$ 18 million. This shows how sensitive is direct cost to coverage, in particular the number of insulin or oral medication users.

**Table 2:** Direct cost of diabetes mellitus (DM) by in Latin America and the Caribbean (LAC) sub–region, 2015

Item	Latin America		English Caribbean		Total	
	**S1**	**S2**	**S1**	**S2**	**S1**	**S2**
**Medication:**						
No. of insulin users (×10^3^)	3869	6550	184	364	4054	6915
No. of oral medication users (×10^3^)	18 851	32 046	898	1 527	19 749	33 573
Cost of insulin (US$×10^6^)	6671	10971	324	640	6995	11611
Cost of oral medication (US$×10^6^)	3811	6479	218	370	4029	6849
Total cost of medication (US$×10^6^)	10 482	17 450	542	1 010	11 024	18 460
**Cost of exams** (US$×10^6^)	1252	2584	128	274	1380	2859
**Consultations:**						
Total no. consultations (×10^3^)	266 169	321 546	13 538	15 605	279 708	337 151
DM related causes (×10^3^)	101 523	156 899	3 789	5 856	105 312	162 755
Cost DM related causes (US$×10^6^)	4872	6536	196	270	5068	6806

The total number of hospitalization (**Table 3**) was 36 746 from which more than 18 thousand were estimated to be attributed to diabetes causing a burden of more than US$ 10 billion. The estimated number of emergency visit was more than 95 thousand with an estimated cost of more than US$ 1 billion.

**Table 3:** Cost of hospitalizations and emergency visit among those with diabetes in Latin America and the Caribbean in 2015

Item	Latin America	English Caribbean	Total
Total No. of hospitalization (×10^3^)	34 747	1998	36 746
Diabetes–related causes (×10^3^)	17 462	832	18 294
Total days	135 834	7 843	143 677
No. days (DM related causes)	66 691	3177	69 868
Cost DM related (US$×10^6^)	10 025	309	10 334
No. emergency visits	91 277	4349	95 626
Cost (US$×10^6^)	1036	22	1059

DM – diabetes mellitus

More than 4 million people with diabetes were estimated to have retinopathy and cardiovascular diseases respectively, while more than 3 million were estimated to have neuropathy and more than 2 million were estimated to suffer from nephropathy or peripheral vascular disease, respectively (**Table 4**).

**Table 4:** Estimated number (×10^3^) of people with diabetes and chronic complications in Latin America and the Caribbean, 2015

Complications	Latin America	English Caribbean	Total
No. person with retinopathy	4671	223	4894
No. person with cardiovascular disease	4496	214	4711
No. person with nephropathy	2306	110	2416
No. person with neuropathy	3421	163	3584
No. person with peripheral vascular disease	2254	107	2362

Treating chronic complications caused an estimated burden between US$ 16.2 and US$ 26.5 billion for S1 and S2 respectively. Cardiovascular disease was the costliest complication with an estimated burden between US$ 5.6 and US$ 8.2 billion followed by retinopathy (US$ 3.5 to US$ 6.4 billion), nephropathy (US$ 2.9 to US$ 4.6 billion), neuropathy (US$ 2.1 to US$ 3.7 billion) and peripheral vascular disease (US$ 2 to US$ 3.4 billion) (**Table 5**).

**Table 5:** The cost (US$×10^6^) of diabetes complications in Latin America and the Caribbean, 2015

Cost	Latin America		English Caribbean		Total	
	**S1**	**S2**	**S1**	**S2**	**S1**	**S2**
Cost of retinopathy	3345	6097	166	347	3511	6444
Cost of cardiovascular disease	5435	7870	235	396	5670	8266
Cost of nephropathy	2755	4434	147	258	2902	4692
Cost of neuropathy	2028	3560	107	208	2134	3768
Cost of peripheral vascular disease	1949	3686	90	1891	2040	3418
Total cost of complications	15 511	25 204	746	1 384	16 257	26 588

Among countries of LAC (**Table 6**) the highest direct cost was estimated for Brazil (between US$ 17.5–US$ 23.8 billion); while the lowest was estimated for Grenada (between US$ 4.0–US$ 6.6 billion). The highest per capita cost was estimated for Puerto Rico, US$ 2764–4949 and the lowest for Peru US$ 445–821 (S2).

**Table 6:** Total and per capita direct cost of diabetes mellitus (DM) and national health expenditures by country and GDP group, Latin America and the Caribbean, 2015

Lower–middle Income	National Health Expenditures (US$)	S1 (Lower)		S2 (Higher)	
**Direct costs (US$×10^6^)**	**Per capita costs (US$)**	**Direct costs (US$×10^6^)**	**Per capita costs (US$)**
Bolivia	420	263.4	677	483.8	1244
El Salvador	565	353.2	1084	665.0	2040
Guatemala	462	1054.3	1385	1943.1	2552
Guyana	361	26.9	540	44.9	901
Honduras	428	464.4	1388	855.9	2559
Nicaragua	445	301.1	1091	512.0	1856
Paraguay	874	301.8	931	542.0	1673
**Sub–total**	508†	2765.1	1014†	5046.7	1832†
**Upper–middle income:**					
Argentina	862	1741.1	1010	3026.3	1756
Belize	487	20.9	728	32.5	1133
Brazil	1318	17 492.5	1227	23 825.7	1672
Colombia	962	2928.3	961	5367.5	1761
Costa Rica	1390	292.1	1047	521.6	1870
Cuba	2280	562.4	551	937.7	919
Dominican Republic	581	317.2	627	470.6	930
Ecuador	1042	1144.8	1379	1753.1	2112
Grenada	758	4.0	580	6.6	957
Jamaica	476	311.7	1538	509.3	2513
Mexico	1091	10 659.1	930	14 246.8	1243
Panama	1678	361.7	1568	605.8	2627
Peru	656	560.7	455	1010.7	821
Saint Lucia	721	8.3	625	13.9	1044
Suriname	947	26.2	625	41.9	1001
Venezuela	950	1773.6	831	2865.3	1343
**Sub–total**	1012†	38 204.6	918†	55 235.3	1481†
**Higher–income:**					
Bahamas	1818	48.5	1345		2021
Barbados	1198	31.7	928	50.8	1487
Chile	1717	1354.9	987	2054.2	1496
Puerto Rico*		1009.1	2764	1806.6	4949
Trinidad & Tobago	1822	190.2	1356	289.4	2063
Uruguay	1792	97.4	618	129.4	821
**Sub–total**	1669†	2731.8	1333†	4403.3	2140
**Total**	1063†	43 701.5	1088†	64 685.3	1818†

* National Health Expenditures (NHE) figure for Puerto Rico was not available.

†Average.

On average, the annual cost of treating one case was between US$ 1088 and US$ 1818. Countries of LAC expended in health an average of US$ 1063, with the highest among reported by Cuba (US$ 2280) and the lowest by Guyana (US$ 361). On average, NHE were notably lower among low income countries (US$ 508 per capita) and the highest among higher income countries (US$ 1063). Average expending on diabetes was close to average NHE for S1 and notably higher for S2. On average, among lower–middle income countries, NHE (US$ 508) was notably lower than the per capita cost of care for people with diabetes assuming either S1 (US$ 1014) or S2 (US$ 1832)

The total cost of diabetes in LAC was estimated to be between US$ 103 and US$ 124 billion in 2015. The highest cost was estimated for Brazil between US$ 37 and US$ 43 billion while the lowest cost was estimated for Grenada between US$ 39 and US$ 42 million (**Table 7**).

**Table 7:** Direct, indirect and total cost of diabetes mellitus (DM) by country and GDP, Latin America and the Caribbean, 2015

Lower–middle income:	Indirect costs (US$×10^6^)	S1 (Lower)		S2 (Higher)	
**Direct costs (US$×10^6^)**	**Total costs (US$×10^6^)**	**Direct costs (US$×10^6^)**	**Total costs (US$×10^6^)**
Bolivia	358	264	622	485	843
El Salvador	245	355	601	667	912
Guatemala	523	1059	1582	1948	2471
Guyana	44	28	72	46	90
Honduras	180	467	648	859	1039
Nicaragua	110	307	417	518	628
Paraguay	229	305	534	546	774
**Sub–total**	1690	2786	4476	5068	6757
**Upper–middle income:**					
Argentina	1740	1771	3511	3057	4796
Belize	21	22	42	33	54
Brazil	19 052	18 271	37 323	24 605	43 656
Colombia	2805	3087	5892	5527	8331
Costa Rica	286	298	584	527	814
Cuba	1590	581	2170	956	2545
Dominican Republic	464	328	792	481	946
Ecuador	742	1187	1929	1795	2537
Grenada	35	4	39	7	42
Jamaica	239	328	567	526	765
Mexico	17 240	10 835	28 075	14 423	31 663
Panama	344	364	708	608	952
Peru	1209	571	1780	1021	2230
Saint Lucia	11	9	20	14	26
Suriname	51	27	78	43	94
Venezuela	6062	1800	7862	2891	8953
**Sub–total**	51 890	39 483	91 373	56 514	108 404
**Higher–income:**					
Bahamas	70	50	120	75	145
Barbados	40	33	73	52	92
Chile	2224	1430	3654	2129	4353
Puerto Rico	440	1035	1475	1832	2272
Trinidad & Tobago	315	199	514	298	613
Uruguay	512	105	617	137	649
**Sub–total**	3601	2852	6453	4523	8125
**Total**	57 181	45 121	102 302	66 105	123 286

**Table 8** presents a summary of detailed explanations of all components, sources of the data, estimations and the corresponding cost result.

**Table 8:** The cost of diabetes in Latin American and the Caribbean in 2015

Indirect cost	Source	Cost (US$)
Number of people with DM	41 576 396. Estimated by the Diabetes Atlas	
Permanent disability	Number of persons with diabetes with permanent disability: 1 043 128. Estimated by multiplying the economically active diabetes population in each country by the proportion of people with permanent disability by age and gender. These proportions were calculated by estimating the proportion of people receiving benefits due to diabetes by age and gender in Brazil, adjusted by diabetes duration and the prevalence of chronic disabling diabetes complication in the Chilean database (those with amputations, Chronic Kidney Disease (CKD), blindness, cerebrovascular disease and infarctions), approximate 33.5% of the total population with diabetes.	
	Cost calculated by multiplying years lost to disability by annual country GNP per capita. Cost of permanent disability in one year.	16 204 846 613
Temporary disability	The estimated number of person with diabetes in the labor force was estimated by discounting the unemployed (according to each country unemployment rate published by the World Bank) among those in working ages (20–64 y of age): 31 872 702. Number of sick days related to diabetes 10.5 days per person per year as per results of the ViCen survey.	
	Number of years lost due sick days: 858 154. Cost calculated by multiplying years of productivity lost by GNP per capita.	13 391 123 563
Mortality	Estimated number of all causes deaths: 1 386 513. Calculated by the Population Attributable Fraction using OR obtained from the BES study.	
	Estimated number of deaths among those with diabetes: 318 102 (173 532–461 789).	
	Estimated number of years lost due to premature mortality: 2 074 633. Years lost before age 65 y with a 3% discount rate. Cost calculated by multiplying years lost to premature mortality in each country by annual GNP per capita.	27 585 053 690
**Indirect cost total**		57 181 023 866
**Direct cost**		
Insulin	Scenario 1: Proportion of people with diabetes using insulin: 10%.	
	Insulin consumption per year: 10 000 units per person	
	Number of insulin users: 4 053 699. Cost of insulin in each country obtained from the PAHO database.	66 995 221 314
	Scenario 2: Proportion of people with diabetes using insulin: 20%.	
	Insulin consumption per year: 10 000 units per person	
	Number of insulin users: 6 914 458. Cost of insulin in each country obtained from the PAHO database.	11 611 301 234
Oral medication	Scenario 1: Oral medication consumption per year: 1500 tablets of Metformin. Users 50% of the diabetes population	
	Oral medication users: 19 748 788. Cost of metformin in each country obtained from the PAHO database.	44 028 574 000
	Scenario 2: Oral medication consumption per year: 1500 tablets of Metformin. Users 80% of the diabetes population	
	Oral medication users: 33 572 940. Cost of metformin in each country obtained from the PAHO database.	6 848 575 800
Consultations	Scenario 1: Estimated that 50% of people were followed by health services. Estimated number of consultations related to DM per year for people with diabetes: 105 312 278. Number of consultations per inhabitant from PAHO’s basic indicators. The ViCen survey indicated that people with diabetes had 3.5 more visits than those without diabetes. Visit for general causes were subtracted. The number of consultations due to diabetes was multiplied by the estimated number of people with diabetes in each country. The cost of consultations per country was obtained from the PAHO database.	5 067 964 481
	Scenario 2: Estimated that 85% of people were followed by health services. Estimated number of consultations related to DM per year for people with diabetes: 162 755. Number of consultations per inhabitant from PAHO’s basic indicators. The ViCen survey indicated that people with diabetes had 3.5 more visits than those without diabetes. Visit for general causes were subtracted. The number of consultations due to diabetes was multiplied by the estimated number of people with diabetes in each country. The cost of consultations per country was obtained from the PAHO database.	6 805 773 280
Hospitalizations	Scenario 1–2: Total number of DM hospitalizations per year for people with diabetes: 18 293 614. The ViCen survey indicated that people with diabetes were hospitalized 7 times and hospital stay was 1.9 d longer than those without diabetes. Number of hospital discharges per inhabitant for the general population from the PAHO basic indicator database was used to estimate the excess hospital days due to diabetes in each country. Cost of one hospital day in each country obtained from the PAHO database.	10 333 828 105
Emergency visits	Scenario 1–2: Total number of DM emergency visit per year for people with diabetes: 95 625 710; estimated number of diabetes related emergency visits 95 626. The ViCen survey indicated that people with diabetes had 1.4 more emergency visit than those without diabetes. Number of emergency visits per inhabitant for the general population from the PAHO basic indicator database was used to estimate the excess emergency visits due to diabetes in each country. Only visits assumed to be related to diabetes were included in cost calculation. Cost of an emergency visit in each country obtained from the PAHO database.	11 058 606 746
Test and laboratory exams	Scenario 1: Includes the cost of one A1c, one lipid profile, one albuminuria test, one EKG, and one X Ray for 50% of the diabetes population of each country. Cost of items in each country obtained from the PAHO database.	1 379 772 482
	Scenario 2: Includes the cost of three A1c, one lipid profile, one albuminuria test, one EKG, and one X Ray for 50% of the diabetes population of each country. Cost of items in each country obtained from the PAHO database.	2 858 452 553
Excess cost of complications	Scenario 1: The age–and–gender weighted probability of diabetes complications (cardiovascular disease 0.11, nephropathy 0.06, neuropathy 0.09, peripheral vascular disease 0.06 and retinopathy 0.12) was obtained from the Chilean QUALIDIAB database. The excess cost of each diabetes complication was applied to the cost of uncomplicated cases following results from the Mexican NIH database (excess cost of complications: neuropathy 1.08; peripheral vascular disease 11.06; cardiovascular disease 1.23; nephropathy 11.76; retinopathy 1.09)	16 256 804 668
	Scenario 2: The age–and–gender weighted probability of diabetes complications (cardiovascular disease 0.11, nephropathy 0.06, neuropathy 0.09, peripheral vascular disease 0.06 and retinopathy 0.12) was obtained from the Chilean QUALIDIAB database. The excess cost of each diabetes complication was applied to the cost of uncomplicated cases following results from the Mexican NIH database (excess cost of complications: neuropathy 1.58; peripheral vascular disease 1.81; cardiovascular disease 1.86; nephropathy 2.96; retinopathy 1.86)	26 587 999 416
**Direct cost total**	Scenario 1	45 120 771 799
	Scenario 2	66 104 537 136
**Total Cost of DM**	Scenario 1	102 301 795 665
	Scenario 2	123 285 561 002

DM – diabetes mellitus, y – year

## DISCUSSION

The estimated number of people with DM in countries of LAC soared from 15 million in 2000 to 41 million in 2015, – a 2.7–fold increase in 15 years. In 2015, the economic burden of diabetes was estimated to be between US$ 103 and US$ 124 billion, which represents a 6 to 7–fold increase in the total cost, 4 to 6–fold increase in direct medical, as well as a 8–fold increase in indirect costs compared to the previously estimated costs for 2000 [1].

Data presented here tends to be conservative and may underestimate the real cost of diabetes. This analysis included the best information available, however, there is a lack of information on the cost of procedures and services. Therefore, these costs were not included in the amount attributed to consultations, emergency visits, hospitalizations or visits to ophthalmologists. On the other hand the frequency of testing and consultations, may vary widely among countries. The cost of medications and care might have variations depending on purchasing capacity and government policies. Because of that, two different scenarios were prepared. For S1 and S2, lower and higher coverage for medication, testing and service as well as cost for treating diabetes complications respectively were assumed. These different estimates resulted in very different direct cost estimates for scenario 1 and scenario 2. Keeping constant indirect costs, the observed variation of the total cost of diabetes reflects the sensitivity to different assumption for each component such as medication and care for complications. Scenario 2 is 31 percent costlier than scenario 1. Sensitivity is also clearly related to the cost of medication. For example If all other cost assumptions are kept as established for S1 and S2, and the cost of insulin is reduced to US$ 4.20 per vial, it would result in cutting direct cost in 50% and the per capita cost of diabetes care by 35% (data not shown).

The proportion of direct and indirect cost was 44% and 56% for S2 and 54% and 46% for S1 respectively. These variations are driven by the difference in access to care and the cost of complications assumed for S1 and S2. These results are consistent with previous research, for example, an African study calculated direct medical cost at 43% and indirect cost at 56% [25] similar to our proportion in S1; while the ADA estimates indicated a higher proportion of direct cost at 72% and lower proportion of indirect cost at 28% [26] because of the well–known high cost of many medical services and procedures in the United States. The Brazilian study estimated the direct and indirect cost at 63% and 37%, respectively [17]. But the difference in the proportion of direct and indirect cost can be also due to the inclusion of different items in the study, real differences in the cost of procedures and medications, as well as differences in access to care.

On average, health care cost was estimated at US$ 1088–1819 per capita with wide ranges from US$ 4979 (S2) in Puerto Rico to US$ 540 (S1) in Guyana. The ADA [26] study estimated the per capita cost of diabetes at US$ 7900 [21] in the US, which was much higher than the cost reported in this study for LAC. In Africa, this figure varied from US$ 2144.3 to US$ 11 431.6 depending on the country’s Gross National Income [25]. Interestingly, there were substantial differences in the items included in the North American, African and this current LAC study. The ADA [26] and the African [25] studies both included diabetes supplies, in addition the ADA [26] study included home care; those items were not included in the present study.

Our results for per capita direct cost was consistent to previous research in the region such as those published for Brazil [17], Argentina [27], and the South–Central America (SACA) Region [27]. The Brazilian study estimated the direct per capita cost to be US$ 1334 [17], which is close to the US$ 1227–1 672 we estimated for Brazilian persons with diabetes. The Argentine study [27] estimated the quarterly direct per capita cost of DM in a social security institution at US$ 904.60 and US$ 1841.80 Argentine pesos for DM cases with and without chronic complications, respectively. These figures translate into US$ 1796.82 (without chronic complications) and US$ 2037.89 (with chronic complications) per year. These estimations are closed to estimations reported in this study for Argentina (US$ 1010–1773) [27]. De Rocha Fernandes et al. [28] calculated health care cost for the IDF–SACA region between US$ 1155 and US$ 2024 for 2014, which overlap to our results for the same region of IDF at US$ 1136–1711 (data not shown).

A big gap was observed between NHE and the cost of diabetes care especially among lower income countries. This gap, as suggested by Arredondo et al [29] in Mexico, may result in out of pocket expenses in some cases [25] or the avoidance of necessary services because of economic constrains in others.

The total economic burden of diabetes among countries is highly driven by the size of the population, baring higher cost for countries with bigger population size such as Brazil, Mexico and Argentina. Per capita direct cost however, depends more on national prices of goods and services. Some of them have proven to be highly variable. The best example is the price of a vial of insulin, which price varied from US$ 78 in Puerto Rico to US$ 1.25 in Cuba (average price US$ 20). The Pan American Health Organization Strategy Fund (PAHO–SF) offers high quality medicine at affordable prices to countries of the Americas [30]. For example, a vial of 100 ml of high quality insulin is offered by PAHO–SF at US$ 4.20 to Member States. Running our analysis replacing country specific prices for insulin and oral medication with PAHO–SF’s prices (plus 15%) in our data, resulted in cutting direct cost in 50% and the per capita cost of diabetes care by 35% (data not shown).

Prevalence estimates in this paper are based on epidemiological studies on the frequency of DM prepared for the Diabetes Atlas [2]. It was assumed that 55% (S1) or 85% (S2) of the total number of cases received care; however, this proportion may vary widely from country to country. Furthermore, the proportion of cases receiving treatment may vary according to access to care, patient’s educational level, and the capacity of the health system.

Major limitations of this study include the lack of information on many issues which were replaced by assumptions that in many cases cannot be confirmed. That is the case of the proportion of cases receiving services, the prevalence of diabetes chronic complications (approximations are based on one local study), and the proportion of people with permanent or temporary disability. In every case the best information available was used to generate estimates for the missing information. The results of this study were consistent when compared, as discussed previously, to small number of research reports available in the medical literature on the subject from Argentina [27], Brazil [17] and the SACA Region [28]. It is, however, suggested that these results be considered with caution, since inaccuracy may occur when making similar assumption for many countries with different health and financial systems. All cost components were adjusted to the age–and–sex population estimates for each country, except for the proportion of cases receiving treatment or under care. The latest was not possible because of the lack of information on these issues. Finally, we used in our estimates commercial prices for services and medication for each country. This was done so because prices for government provided goods and services were not available in every country. It may be possible that these services and medications are available at lower subsidized prices in some of the countries. Prices of medication and services for each country depended on information provided by a wide variety of collaborators including government officials, member of diabetes or professional associations, health providers and other volunteers. Biases introduced by the observers could not be controlled in any possible way.

While providing comparable estimates of the cost of diabetes among countries, this paper alerts public health officials of the growing burden of DM in LAC. It provides arguments to reinforce needs to strengthen public policies that promotes health and diabetes prevention, such as those that discourage the consumption of junk food or sugary drinks and promote physical activity; as well as those focused on increasing access to diabetes care and supplies.

DM represents a major public health problem in LAC. Middle and low–income countries face a major challenge that not only affects their health system organization and financing, but also their economic growth. Buying medication from PAHO–SF instead of paying market prices may reduce the burden that diabetes care represents to countries and individuals. As the prevalence of DM is expected to increase in the near future, so will its toll on individuals, families and society as a whole.

## CONCLUSIONS

The high economic burden of diabetes showed here has strong implications for the health systems and the economic grow of the nations of LAC. This burden is related to direct medical care but also to indirect cost caused by loss of productivity due to premature death and disability. Multi–sector public policies could contribute to curve this treat on the long term.
