# Supplementary material for: The cost of diabetes in Latin America and the Caribbean in 2015: Evidence for decision and policy makers
Source: J Glob Health. 2017 Nov 10;7(2):020410. doi: 10.7189/jogh.07.020410 (PMC5681710; doi:10.7189/jogh.07.020410)
Supplement: Online Supplementary Document [file jogh-07-020410-s001.pdf]

Online Supplementary Document

Barcelo et al. The cost of diabetes in Latin America and the Caribbean in 2015: Evidence for decision and policy makers

J Glob Health 2017;7:020410

Table S1. List of country specific medications, testing and services

	INSULIN	METFORMIN	A1C	Lipid Profile	EKG	RX	Urine Protein	Consultation	Emergency Visit	Hospitalization	Visit ophthalmologist
Argentina	$26.25	$0.11	$5.20	$7.12	$11.51	$7.53	$4.07	$29.17	$29.17	$90.03	$40.17
Barbados	$17.41	$0.05	$22.50	$7.12	$7.53	$11.51	$2.52	$35.86	$35.86	$110.68	$49.39
Belize	$3.73	$0.01	$11.09	$17.65	$6.07	$14.59	$0.89	$16.07	$16.07	$96.31	$24.38
Bolivia	$15.49	$0.11	$15.40	$16.80	$35.00	$9.80	$4.07	$21.00	$9.40	$8.40	$28.00
Brazil	$10.34	$0.14	$6.40	$12.80	$9.60	$12.80	$12.80	$48.00	$96.00	$256.00	$64.00
Chile	$21.70	$0.01	$5.27	$7.12	$7.53	$11.51	$0.98	$55.08	$55.08	$148.47	$58.67
Colombia	$26.00	$0.15	$15.70	$13.00	$14.10	$51.99	$2.49	$10.30	$16.89	$110.58	$14.85
Costa Rica	$22.00	$0.22	$12.54	$12.71	$39.94	$38.00	$6.66	$23.05	$23.05	$71.14	$31.75
Cuba	$1.25	$0.20	$25.00	$20.00	$30.00	$25.00	$3.62	$14.92	$14.92	$46.04	$20.55
Dominican R	$7.00	$0.02	$20.00	$42.00	$20.00	$16.00	$13.00	$13.00	$13.00	$117.00	$13.00
Ecuador	$22.00	$0.10	$12.00	$15.00	$5.00	$5.08	$1.96	$60.00	$60.00	$180.00	$60.00
El Salvador	$23.25	$0.25	$29.90	$40.00	$30.00	$20.00	$60.00	$15.00	$11.95	$30.00	$40.00
Guatemala	$33.00	$0.28	$18.00	$23.00	$25.00	$35.00	$42.00	$26.00	$129.00	$39.00	$45.00
Guyana	$12.05	$0.01	$19.80	$22.28	$7.42	$7.42	$1.67	$12.26	$12.26	$73.56	$12.26
Honduras	$33.00	$0.28	$18.00	$29.78	$31.74	$35.00	$42.00	$26.00	$129.00	$39.00	$45.00
Jamaica	$34.50	$0.09	$34.80	$17.40	$10.44	$34.80	$2.12	$17.40	$34.80	$304.50	$32.00
Mexico	$20.20	$0.14	$10.15	$14.33	$7.53	$11.51	$4.00	$26.79	$26.79	$82.66	$36.89
Nicaragua	$20.00	$0.17	$22.00	$20.00	$20.00	$40.00	$7.50	$30.00	$30.00	$100.00	$30.00

Panama	$12.00	$0.56	$15.00	$25.00	$12.00	$12.00	$35.00	$60.00	$60.00	$75.00	$60.00
Paraguay	$26.00	$0.14	$5.52	$6.63	$4.41	$3.00	$1.98	$15.60	$15.60	$93.41	$30.00
Peru	$7.20	$0.12	$22.80	$8.23	$4.80	$5.44	$16.00	$3.20	$6.40	$49.51	$4.80
Puerto Rico	$77.95	$0.25	$25.00	$40.00	$50.00	$40.00	$40.00	$68.95	$68.95	$212.79	$94.96
Suriname	$9.15	$0.09	$7.98	$10.79	$10.90	$3.63	$2.28	$22.11	$22.11	$68.23	$30.45
Trinidad & T	$23.85	$0.04	$5.27	$7.12	$11.51	$7.53	$2.89	$62.54	$62.54	$156.34	$93.80
Uruguay	$6.19	$0.02	$2.66	$1.28	$1.08	$0.84	$0.81	$36.97	$36.97	$114.09	$50.91
Venezuela	$17.41	$0.05	$5.27	$7.12	$11.51	$7.53	$2.52	$28.82	$28.82	$88.94	$39.69
